# Intracranial Hemorrhage Secondary to Vaping: A Case Report and Literature Review

**DOI:** 10.7759/cureus.40288

**Published:** 2023-06-12

**Authors:** Arielle H Aiken, Ariana R Tagliaferri, Mark Conforti, Roshni Khilnani

**Affiliations:** 1 Internal Medicine, St. Joseph's Regional Medical Center, Paterson, USA; 2 Internal Medicine, Rowan University School of Osteopathic Medicine, Stratford, USA; 3 Psychiatry, Bergen New Bridge Medical Center, Paramus, USA

**Keywords:** intracranial bleeding, intracerebral hemorrhage, cannabis, e-cigarette, vaping

## Abstract

Vaping and marijuana use are becoming more common and accessible in young adults. However, questions remain regarding their long-term health implications. Current literature does not suggest that vaping causes intracranial hemorrhages. Here, we present a case of a 32-year-old male with no medical history other than vaping and marijuana use, who was found to have an acute intraventricular hemorrhage presenting as syncope. This paper explores the relationships between vaping, marijuana use, and strokes in the general population, and further elaborates on the effects of vaping in young adults. We hope to raise awareness of the negative health implications vaping has on otherwise healthy patients and encourage clinicians to take thorough histories and identify those who vape so that comprehensive education can be provided.

## Introduction

Electronic cigarettes, also known as e-cigs, vape pens, e-hookahs, or mods, are devices that enable the inhalation of an aerosol containing nicotine, flavored solutions, tetrahydrocannabinol (THC), and other additives [1]. The solvent in which the nicotine is dissolved consists of propylene glycol and glycerin, which are aerosolized by heat [1]. Depending on the temperatures at which the solvent is heated, high levels of harmful carcinogens, such as formaldehyde, can be released [1]. Since 2018, 8.1 million adults, one in 20 middle school students, and one in five high school students use e-cigarettes [1,2]. While e-cigarettes were initially marketed in the United States in 2010 as a means of reducing tobacco use, it has since been associated with several negative short- and long-term health implications [2]. A plethora of e-cigarette-related lung diseases have been described, including acute respiratory distress syndrome (ARDS), eosinophilic and organizing pneumonia, e-cigarette-associated lung injury (EVALI), and diffuse alveolar hemorrhage [3]. However, data supporting the neurovascular complications of e-cigarette use is limited.

Current literature suggests that e-cigarette use causes an up-regulation of the pro-inflammatory cascade across the blood-brain barrier [4]. Tobacco use alone is a known risk factor for strokes, but recent studies have demonstrated a correlation between strokes and marijuana and e-cigarette use as well [5,6]. One study found that the adjusted odds of developing any stroke, particularly ischemic strokes, were higher among marijuana users compared to non-users [7]. Additionally, the use of concomitant e-cigarettes and marijuana is associated with 2.9 times higher odds of stroke compared to the general population, but there is no increased risk of stroke in patients who only smoke e-cigarettes [8]. However, it has been shown that e-cigarette use is associated with an earlier onset of stroke compared to traditional tobacco users [9].

As the incidence of stroke is rapidly rising among the youth and is now the ninth leading cause of death among 25- to 44-year-olds, education on smoking cessation, particularly with e-cigarettes and marijuana, is of utmost importance [10]. We present the case of a 32-year-old male with longstanding marijuana and e-cigarette use who was diagnosed with a large intraventricular hemorrhage.

## Case presentation

**Figure 1:**
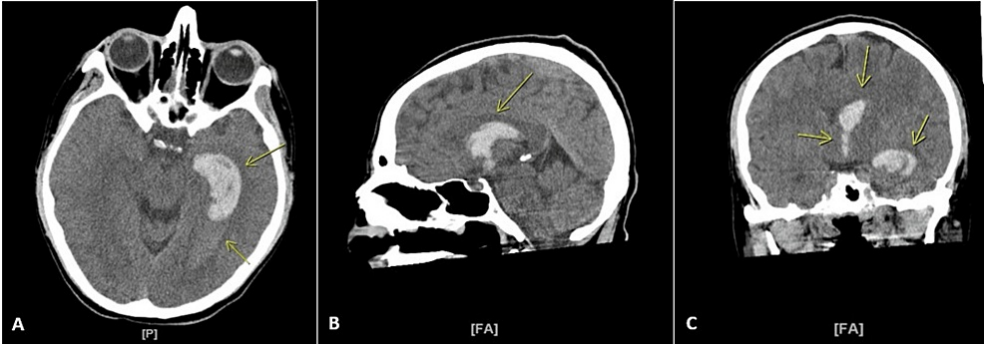
CT of the brain demonstrating acute intraventricular hemorrhage A multi-detector helical head CT was performed without the use of intravenous contrast. A: Axial view with an arrow indicating acute large intraventricular hemorrhage filling the left lateral ventricle and distending the temporal horn B: Oblique sagittal view with an arrow indicating lateral ventricle intraventricular hemorrhage C: Oblique sagittal view with an arrow indicating spillage of the hemorrhagic content into the third ventricle and inferior tip of the lateral ventricle

A 32-year-old Caucasian male with a past medical history of migraines, vaping, and marijuana use, presented to the ED via ambulance after suffering a syncopal episode during sexual intercourse. Further history obtained later revealed the patient reported multiple episodes of vomiting earlier that day, associated with a severe left-sided headache. He has vaped Juul (with 5% nicotine and cool mint flavored) and Lava (with 5% nicotine and banana flavored), and has smoked a pipe of marijuana daily for the last 20 years. On arrival at the ED, he was afebrile and vitally stable with an initial blood pressure of 111/75, a heart rate of 69, a respiratory rate of 17, and an oxygen saturation of 98% on room air. His examination was significant for mild slurring of speech, disorientation to time and place, and minimally decreased visual acuity bilaterally, with no evidence of lateralizing signs or focal neurological deficits. Laboratory analysis was only significant for mild leukocytosis (13 x 10^3/mm3; reference range: 4.5-11) and urine toxicology screen was positive for tetrahydrocannabinol (THC). However, it was negative for amphetamines, cocaine, barbiturates, opiates, and phencyclidine (PCP). A heavy metal screen was not completed. A CT of the brain without contrast revealed acute intraventricular hemorrhage involving both the left lateral and third ventricles with a 6 mm left-to-right midline shift (Figure 1).

**Figure 2:**
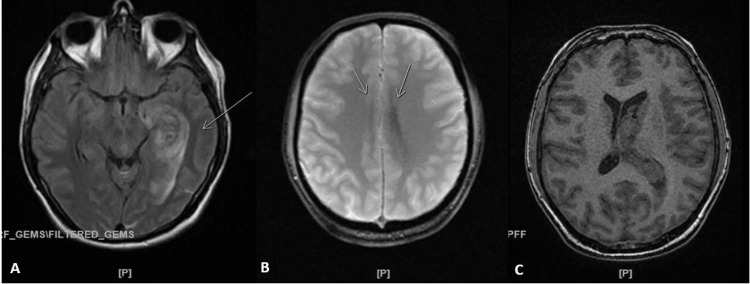
Mri of the brain demonstrating the extension of intraventricular hemorrhage, midline shift, and bilateral subarachnoid hemorrhages A multiplanar/multi-sequence MRI of the brain was performed without contrast. A: FLAIR, axial view. Seen is a re-demonstration of left deep temporal lobe hemorrhage (arrow) extending into the left lateral ventricle and third ventricle B: GRE image, axial view. Arrows indicate bilateral subarachnoid hemorrhage involving both hemispheres up to the high convexity C: T1 axial 3D image. Observed is a midline shift measuring approximately 6 mm FLAIR: Fluid-attenuated inversion recovery, GRE: Gradient echo

A CT angiogram revealed no active hemorrhage, aneurysm, stenosis, or venous thrombosis. An MRI of the brain without contrast revealed similar findings of extensive hemorrhage in the left temporal lobe extending to the left lateral ventricle and third ventricle with interval development of the lateral hemispheric subarachnoid hemorrhage and a slight midline shift with no aneurysm or vascular malformation appreciated (Figure 2).

Magnetic resonance venography and angiography were negative for venous thrombosis, aneurysms, and dissection. Intravenous dexamethasone and levetiracetam were administered, and the patient was admitted to the surgical intensive care unit for close monitoring. Neurosurgery was consulted, and the patient was initiated on twice-daily levetiracetam 500 mg for seizure prophylaxis and nimodipine 60 mg every four hours to achieve a blood pressure of less than 140/90. 

On day two of hospitalization, the patient developed short-term memory loss, mood lability, and expressed suicidality, requiring one-to-one monitoring and psychiatric consultation. At this time, it was thought that the butalbital-acetaminophen-caffeine pill he was receiving for his headaches, coupled with the presence of the intraventricular hemorrhage, was contributing to his delusions and impulsivity. He was then switched to sumatriptan. A transthoracic echocardiogram (TTE) performed to evaluate for embolic sources was negative. Throughout his hospitalization, he continued to have headaches, diplopia, and intermittent fevers. His blood cultures were negative, and repeat brain imaging revealed a stable intracerebral hemorrhage. His febrile episodes were later attributed to autonomic dysregulation. After one week, his delusions and other neurological symptoms improved, and he was discharged home.

## Discussion

Recent studies have demonstrated that strokes occur at an earlier age in patients who use e-cigarettes compared to traditional tobacco use [8,9]. As e-cigarettes are more accessible to children and teenagers, it consequently, increases the risk increases with the extended duration of use [8]. In a study among young adults aged 18 to 44 years old, the risk of stroke using e-cigarettes alone was not increased, however, with concomitant marijuana or prior standard cigarette use, the odds were significantly increased [8].

Studies exploring the physiological and pathological effects of vaping on the neurovasculature are limited [4]. Hedlt et al. demonstrated that e-cigarettes cause a disruption in the integrity of the blood-brain barrier and induce pro-inflammatory cascades due to increased leukocyte-endothelial cell interactions [4]. This was also achieved by the downregulation of several genes, reducing tight junction protein expressions, such as occludin and glucose transporter 1 (GLUT1) [4]. Furthermore, endothelial destruction caused by oxidative stress and lipid peroxidation, as a result of e-cigarette use is reiterated in other studies [11].

While the exact mechanism by which vaping induces intracranial hemorrhages is unclear, previous studies have illustrated aneurysmal ruptures from targeted interactions of nicotine with vascular endothelial cell nicotinic acetylcholine receptors, containing alpha subunits [12]. Additionally, one case report described a female patient with a history of vaping cannabis oil who was diagnosed with reversible vasoconstriction syndrome (RVS) and associated intraparenchymal hemorrhage [13]. Reversible vasoconstriction syndrome commonly affects the temporal lobes, which was a similar finding in our patient, thus a possible differential diagnosis; however, angiographic studies did not reveal the dynamic multifocal segmental cerebral artery vasoconstriction that is typically seen in RVS [13]. Our patient also had no evidence of aneurysmal dilation or active hemorrhaging to suggest aneurysmal ruptures [13]. Thus, the exact mechanism by which our patient suffered an intracranial bleed is unknown. 

## Conclusions

Vaping has adverse health effects on patients of all ages, and its consequences are being discovered every day. Further research is needed to specifically identify the mechanisms behind each pathology associated with vaping so that targeted therapies can be established. Strokes are rarely caused by vaping or marijuana use alone. No case report to date describes an intracranial hemorrhage from vaping in a young adult, specifically with this brand of product. Preventive measures through education and e-cigarette cessation should remain paramount in management strategies. 
